# The pre-activated immune response induced by LPS protects host from leptospirosis

**DOI:** 10.1371/journal.pone.0242742

**Published:** 2020-11-24

**Authors:** Xi Chen, Xufeng Xie, Dianjun Wu, Shilei Zhang, Wenlong Zhang, Yongguo Cao

**Affiliations:** Department of Clinical Veterinary Medicine, College of Veterinary Medicine, Jilin University, Changchun, People’s Republic of China; National Institutes of Health, UNITED STATES

## Abstract

Leptospirosis is an important global zoonosis caused by pathogenic *Leptospira*. It is estimated that more than 1 million people are infected by *Leptospira* each year, and the death toll is about 60,000. Some studies showed that delayed immune response was associated with severe leptospirosis, and TLR4 was very important in the control of leptospirosis. In this study, we aimed to explore the effect of the classical activator (LPS) of TLR4 on leptospirosis in susceptible and resistant hosts. The results showed that LPS pretreatment increased the survival rate of hamsters to 80%. And LPS pre-treatment also significantly reduced the leptospiral load and alleviated the pathological injury in organs of hamsters and mice. The result detected by ELISA in mice showed that the levels of TNF-α and IL-1β were increased in the LPS-treated group compared to the control group before infection. However, two days after infection, the level of cytokines in LPS group was down-regulated compared with that in control group. In addition, *in vitro* results showed that LPS pre-treatment enhanced the phagocytosis and bactericidal ability of macrophages on *Leptospira*. Collectively, our results indicated that the pre-activated immune response induced by LPS enhanced the ability of host against leptospirosis.

## Introduction

Leptospirosis is a widespread zoonosis caused by pathogenic *Leptospira* [1]. It is estimated that more than 1 million people are infected with *Leptospira* each year, and the death toll is about 60,000. Humans and animals can be infected by direct or indirect contact with infected animals or with *Leptospira*-contaminated soil and water [2]. Then, *Leptospira* invade the body through damaged mucosa and skin, and diffuse into internal organs, causing clinical symptoms such as jaundice, liver and kidney damage, pulmonary hemorrhage and even death [3]. Currently, the treatment of leptospirosis mainly depends on antibiotic therapy [4]. However, the early symptoms of leptospirosis are easily confused with other diseases, and the optimal treatment period is often missed [5]. Antibiotics can effectively treat leptospirosis, but improper use of antibiotics can aggravate leptospirosis [6,7]. Besides, antibiotics not only have toxic effects and side effects, but also long-term use of antibiotics may promote bacteria to develop drug resistance. Therefore, it is urgent to explore a new and effective way to prevent and cure leptospirosis.

Leptospirosis is tightly associated with inflammatory storms [8]. However, the therapeutic effect of immunosuppressant did not achieve the desired effect [9]. Combined with the gene expression profiles of golden hamsters and mice, we found that after infection with *Leptospira*, the gene expression of hamster’s lags that of tolerant mice [10]. Therefore, we suspect that early activation of the host immune response can resist leptospirosis.

Toll-like receptors (TLRs) play important roles in resisting pathogen [11]. In tolerant animals, macrophages recognize *Leptospira* through TLR2 and TLR4 [12]. And TLR4-deficient tolerant animals showed severe pathological changes after infection with *Leptospira* [13]. Thus, activating host TLR4 may be the key to the treatment of leptospirosis. Lipopolysaccharide (LPS) is a component of the outer wall of Gram-negative bacteria, and it is the classical ligand of TLR4 [14]. Therefore, we speculate that LPS can effectively protect the host from leptospirosis.

In this experiment, we found that LPS plays an effective role in the preventive of leptospirosis. The survival rate of hamsters pre-treated with LPS was significantly increased. In addition, the load of *Leptospira* and the degree of pathological injury of kidney, liver, lung significantly decreased compared with the control group. In addition, LPS pre-treatment enhanced the phagocytosis and bactericidal ability of macrophages on *Leptospira*. Our study provides a new direction for the prevention of leptospirosis in the future.

## Methods and materials

### Ethics statement

Eight- to ten-week-old mice and four-week-old Syrian golden hamsters (*Mesocricetus auratus*) were provided by the Liaoning Changsheng biotechnology co. LTD. During the experiment, all mice and hamsters were fed with standard feed, free water supply, 12h light/12h dark cycle. All animal experiments are carried out in accordance with the regulations of China on the Administration of Experimental Animals. The protocol was approved by the Animal Protection and Utilization Committee of Jilin University (20170318).

### Bacterial strain

Pathogenic *Leptospira interrogans* serovar Lai strain Lai (56601) was grown in liquid Ellinghausen–McCullough–Johnson–Harris (EMJH) medium at 29°C. And *Leptospira* used in the infection test were cultured for no more than three generations.

### Cell culture and stimulation

Bone marrow macrophages cells (BMDM) derived from mice which were treated with LPS 24 h in advance or the controls were seeded at 10^6^ cells in six-well plates cultured in DMEM media (HyClone, USA) supplemented with 10% fetal bovine serum (FBS; HyClone, USA), 100 U/ml penicillin and 100 μg/ml streptomycin, 20% L929 supernatant in 5% CO_2_ atmosphere at 37°C. On the 7th day, cells were washed with sterilized PBS and then cultured in antibiotic-free medium. One hour after infection with *Leptospira*, the phagocytosis rate of *Leptospira* was detected. BMDM were extensively washed with RPMI to remove extracellular bacteria and then incubated for 1 h in a medium containing gentamicin (25 mg ml^-1^), to kill the remaining extracellular bacteria. Then, the infected BMDM were incubated in a gentamicin-free medium. After four hours, BMDMs were lysed at the indicated times with 1 ml of distilled water and 100 ml aliquots were used to inoculate 2 ml of EMJH broth. The tubes were incubated after 6–7 days in EMJH broth, the number of bacteria was determined with a Petroff-Hauser chamber under dark-field microscopy.

### Experimental infections

Mice (2 μg/100 g) and hamsters (10 μg/100 g) were intraperitoneally injected with LPS (derived from E. coli) prior 2 hours or 24 hours to infection. Then, hamsters were injected intraperitoneally with 10^7^ *Leptospira*, and mice were injected intraperitoneally with 10^8^ *Leptospira*. Four hamsters of each group were humanely euthanized by using CO_2_ and organs (liver, kidney and lung) of hamsters were collected. The organs were stored at -80°C for follow-up detection.

For survival assay, animals were monitored daily for signs of illness including weight loss and mobility loss and were euthanized when they appeared moribund. For the other experiments, hamsters were euthanized at the indicated days. All mice were euthanized the day after infection. Before sacrifice, all animals were anesthetized with isoflurane 3% to alleviate suffering.

### Histopathological examination

Hamster kidneys, livers and lungs were taken and placed in 10% neutral buffer formalin, dehydrated, embedded in paraffin, sliced and stained with hematoxylin eosin. The pathological changes were examined by microscope [15].

### Real-time qPCR (RT-qPCR)

According to the manufacturer’s instructions, use TRIzol (Invitrogen, USA) to extract total DNA from 0.1g of organs. Applied Bioscience 7500 Thermal Cycling instrument and FastStart Universal SYBR GREEN Master (Roche Applied Science, Germany) were used to quantify the DNA concentration of *Leptospira* [16].

### Enzyme-linked immunosorbent assay (ELISA)

The mouse kidneys and lungs were ground in PBS, centrifuged at 4°C and centrifuged by 3000rpm for 10 minutes, then stored in-80 °C, and then cytokines were detected in supernatant using mouse ELISA kit (eBioscience) as instructed by the manufacturer.

### Statistical analysis

The data are presented as mean ± SD. Statistical analyses were performed by One-way ANOVA followed by the Newman–Keuls test. Survival differences between the study groups were compared by using the Kaplan-Meier log-rank test. Differences were considered significant at p < 0.05.

## Results

### Pre-treatment with LPS significantly increased the survival rate of hamsters

**Fig 1:**
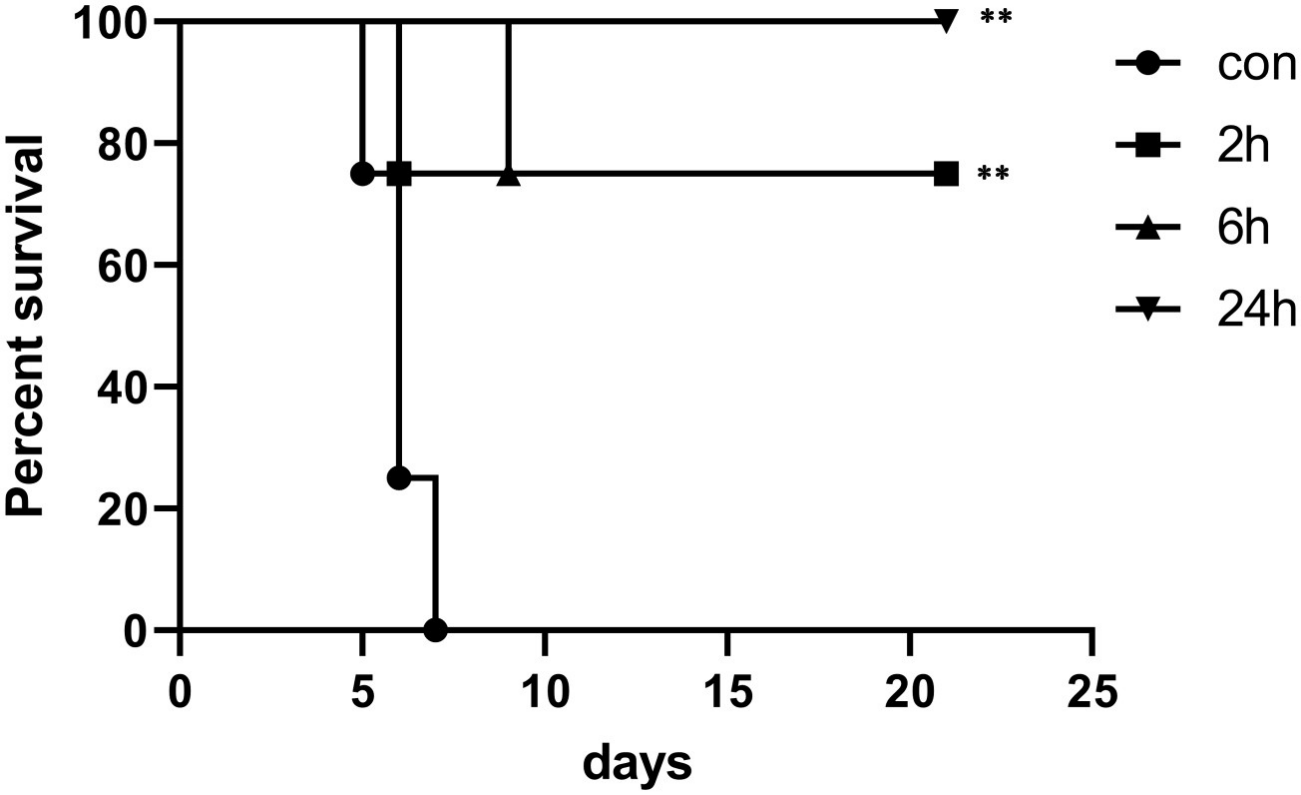
Survival curve of hamsters in infection control group and LPS treatment group. Survival differences between study groups were compared using the log-rank test. *P<0.05, **P<0.01 vs. the control group.

In order to explore the protective effect of LPS on the leptospirosis of hamsters, we injected LPS or normal saline, intraperitoneally to hamsters prior to infection. The results showed that all the hamsters in the control group died within one week (Fig 1). However, the survival rate of LPS pre-treatment hamsters infected with leptospira was increased to 80%. These results suggest that LPS pre-treatment is helpful to resist leptospirosis.

### LPS pre-treatment alleviated pathological damage and reduced *Leptospira* load in hamsters

**Fig 2:**
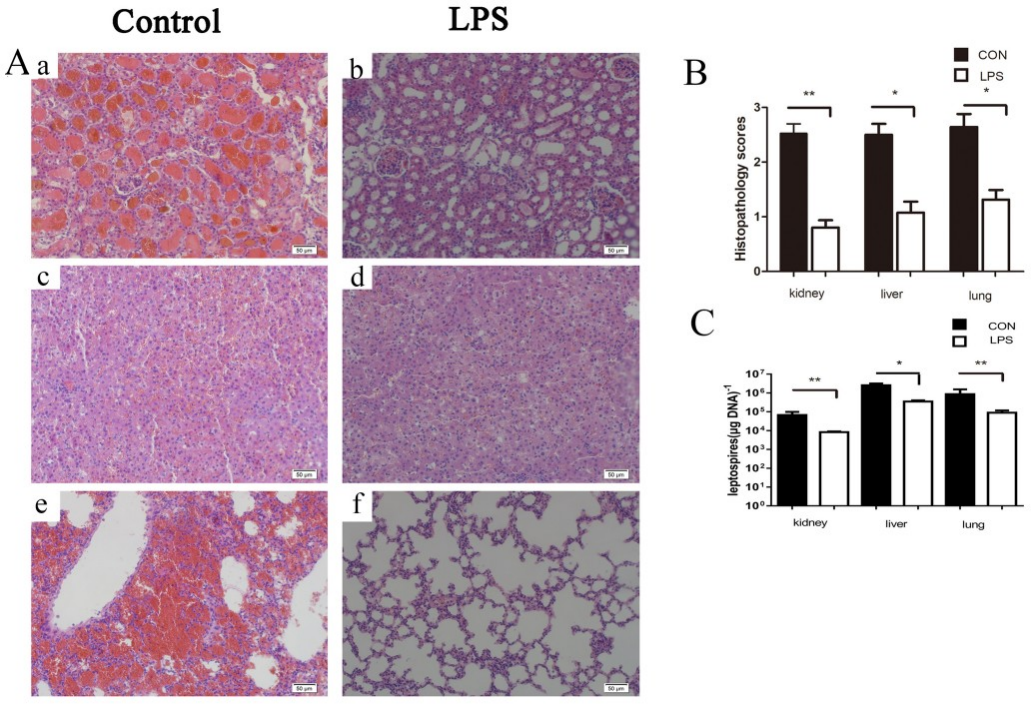
(A) Histopathology of Kidney (a and b), Liver (c and d), and Lung (e and f) of hamsters in the infected control group and the LPS-treated group. Samples were collected at 2 d p.i., and representative photographs are presented (B) Histopathology scores for kidneys, livers, and lungs of hamsters. The data represent the mean histopathology scores for the two groups of hamsters. Statistical analysis of the results for infected controls (n = 3) and the LPS-treated group (n = 3) was performed by using the Wilcoxon rank sum test. *P < 0.05, **P<0.01. (C) Leptospiral burdens in the kidneys, livers, and lungs of hamsters in the infected control group (n = 4) and the LPS-treated group (n = 4) was determined by qPCR. Samples were collected at 2 d p.i. The results are presented as numbers of genome equivalents per microgram of tissue DNA, and the differences were compared by t-test. *P < 0.05, **P<0.01 and ***P<0.001.

As 24 h in advance LPS treatment exhibited the best protective effect, we chose this time point as subsequent experiments. The infection model in Fig 1 was followed. Hemorrhagic damage was obvious in the renal tissue of the infection control group. By contrast, there was little evidence of bleeding in the kidneys of the LPS group (Fig 2Aa and 2Ab). Compared with LPS group, there were more inflammatory lesions in liver tissue and wider intercellular space in infection control group (Fig 2Ac and 2Ad). Severe pulmonary hemorrhage occurred in the infection control group, but no bleeding focus was found in the early LPS injection group (Fig 2Ae and 2Af). The histopathological score (kidneys, livers and lungs) was consistent with the histopathological examination (Fig 2B). The load of *Leptospira* in kidney, liver and lung tissues of mice in control group and LPS group was detected by qPCR. The result showed that the *Leptospira* load in control group was significantly higher than that in LPS group (Fig 2C). The results showed that pre-treatment of LPS could reduce organ injury and *Leptospira* load in golden hamsters.

### Pre-treatment with LPS reduced the load of *Leptospira* in mice

**Fig 3:**
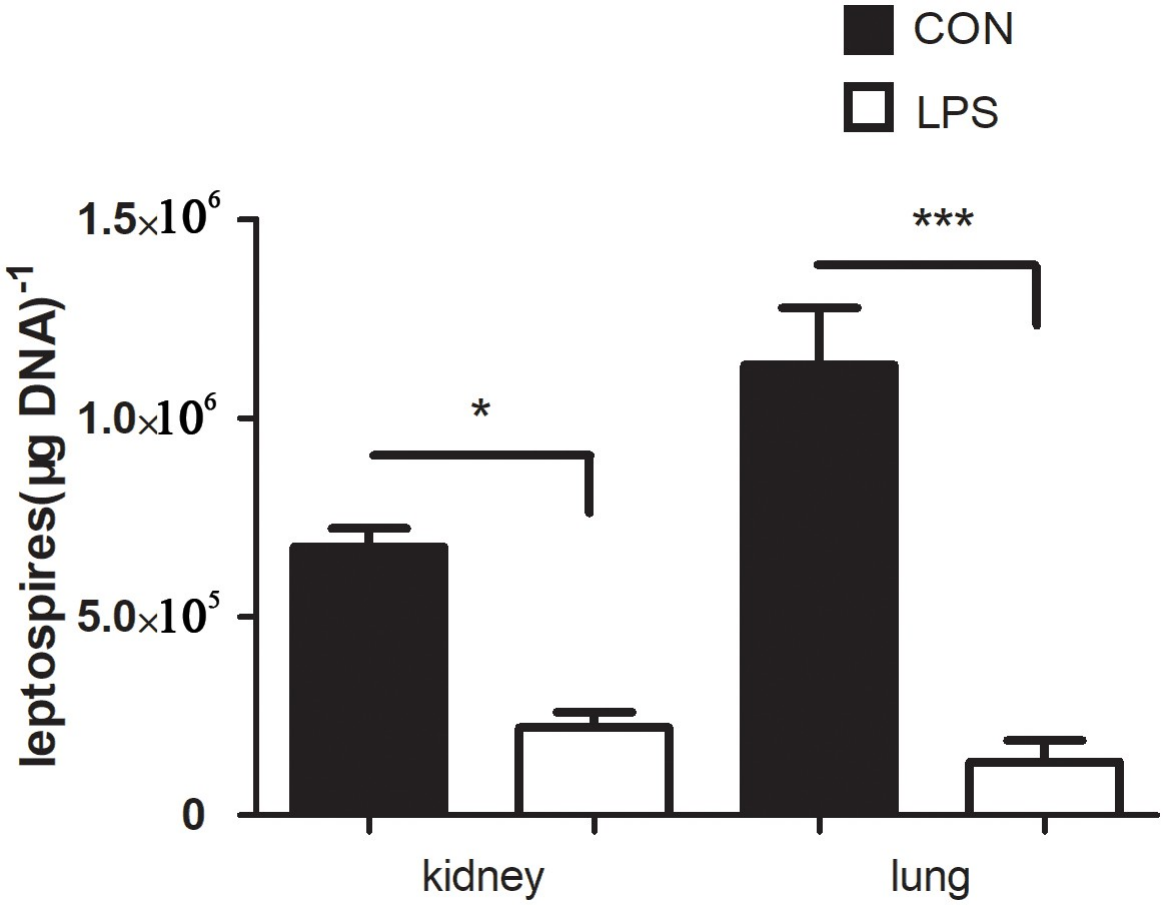
The load of *Leptospira* in organs of mice in infection control group and LPS treatment group on the second day after infection. Values represent the means ± standard errors of the means (SEMs), and differences between the mean values were analyzed by t-test. *p < 0.05, **P<0.01 and ***P<0.001.

In order to verify the anti-leptospirosis effect of LPS in mice, LPS was injected 2 days in advance, and mice were infected with 10^8^ *Leptospira*. The load of *Leptospira* was observed 2 days after infection. The result showed that the *Leptospira* burden in control group was significantly higher than that in LPS group (Fig 3). It is suggested that LPS pre-treatment can also prevent *Leptospira* infection in mice.

### LPS pre-treatment reduced the level of inflammation after *Leptospira* infection

**Fig 4:**
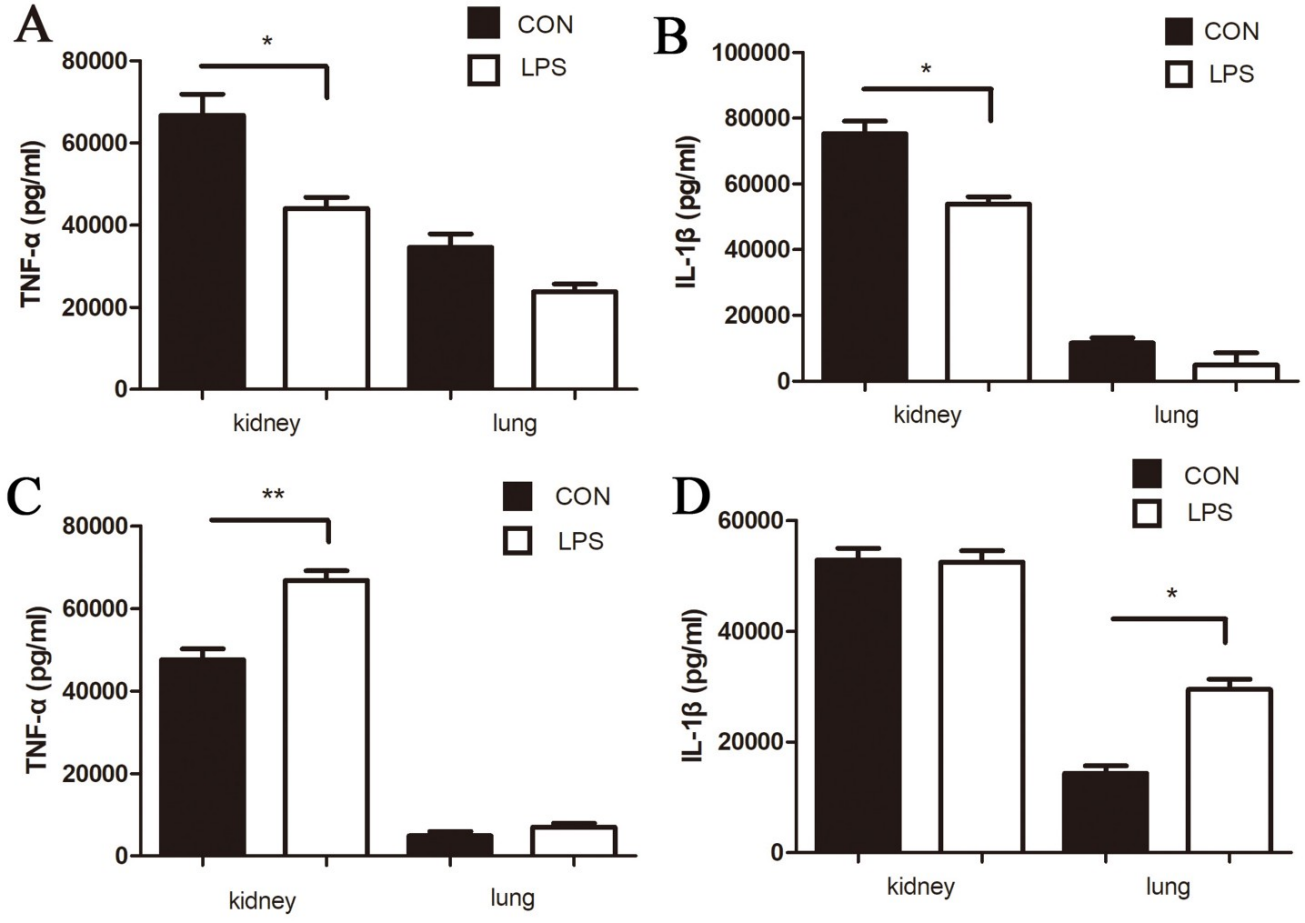
A (TNF-α) and B (IL-1β) are the levels of cytokines in *Leptospira* infection control group and LPS group. C (TNF-α) and D (IL-1β) were the cytokine levels of control group and LPS group without *Leptospira* infection. t-test was used to compare the difference between infection control group and LPS treatment group. * P<0.05, **P<0.01.

In order to verify the effect of LPS on the early inflammatory response induced by *Leptospira*, 2 days after *Leptospira* infection, the samples were collected and detected by ELISA. The protein levels of TNF-α and IL-1β in LPS treated mice were higher than those in the control group before (Fig 4C and 4D). However, after infection with *Leptospira*, the level of inflammation in the LPS group was lower than that in the control group (Fig 4A and 4B).

### LPS pre-treatment promoted the phagocytosis index and bactericidal ability of BMDM on *Leptospira*

**Fig 5:**
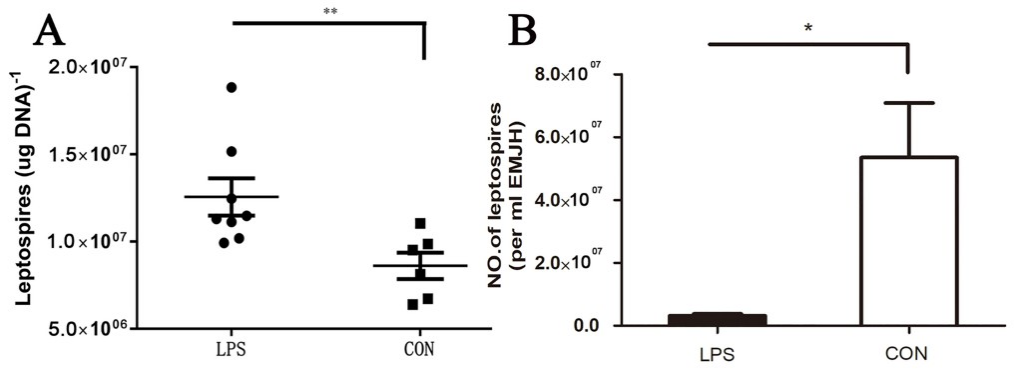
Effect of LPS on bone marrow macrophages (A), After *Leptospira* infected bone marrow macrophages for 1 hour, the extracellular *Leptospira* was removed, and the number of *Leptospira* in the cells was detected by real-time fluorescence quantitative PCR. the differences were compared by t-test. *P < 0.05 **P<0.01. (B) the stock of *Leptospira* in bone marrow macrophages at different times was cultured with EMJH broth and counted by Petroff-Hauser chamber under dark field microscope to quantify the bacteria or cell-associated bacteria in the culture supernatant. The data is the average SD of three independent experiments. t-test was used to compare the difference between infection control group and LPS treatment group. * P<0.05.

In order to elucidate the protective effect induced by LPS, we studied BMDM *in vitro*. The results showed that the number of *Leptospira* phagocytosed by macrophages in LPS group was higher than that in control group (Fig 5A), while the number of remaining surviving *Leptospira* in 4 hours was lower than that in control group (Fig 5B). The experimental results show that LPS pre-treatment enhances the body’s ability to remove *Leptospira*.

## Discussion

Leptospirosis is a zoonotic disease that can infect almost all mammals and cause a wide range of clinical symptoms [1,17,18]. Antibiotic therapy is the main way of treatment at present, however antibiotics can cause toxicity and drug resistance [4,19]. During *Leptospira* infection, the inflammatory response of sensitive animals is delayed [10], Therefore, we speculate that early activation of the immune system is an effective treatment for leptospirosis. In view of the important role of TLR4 in the control of leptospirosis. In view of the important role of TLR4 in controlling leptospirosis [13], so we hypothesized that TLR4 receptor agonist LPS could help control leptospirosis, and tested the efficacy of LPS in the resistance of leptospirosis [13]. The results showed that the inflammatory response induced by LPS was effective in the prevention of leptospirosis.

The delayed inflammatory response in sensitive animals may be the reason for their susceptibility [10]. We used LPS to activate immunity in advance, which greatly improved the survival rate. This coincides with the latest progress in domestication immunotherapy for leptospirosis [20]. The protein levels of pro-inflammatory cytokines TNF-α and IL-1β were up-regulated in mice induced by LPS. It is reported that IL-1β and TNF-α promote the activation of macrophages, and subsequent secretion of immuno-regulators including pro-inflammatory factors that amplify the inflammatory response [8].

Toll-like receptors (TLRs) plays an important role in resisting pathogen [21]. TLR2 and TLR4 can recognize *Leptospira* in mice [12,22], mediate downstream signal pathways, release inflammatory factors, and promote the clearance of *Leptospira* [23–25]. In particular, TLR4 has played an important role [13]. TLR4 deficiency causes death in mice infected with *Leptospira*. Gram-negative bacteria LPS is the most characteristic ligand of TLR4 [14]. In the experiment, we found that LPS pre-treatment can effectively prevent *Leptospira* infection. Therefore, targeting TLR4 may be an effective way to against leptospirosis in the future.

Interestingly, in the late stage of leptospirosis, inflammatory storm causes death of the body [8]. Our experimental results showed that the inflammatory level of mice infected with *Leptospira* did not increase sharply after LPS pre-treatment, which made mice avoid being damaged by their own excessive inflammatory response after infection with *Leptospira*. The decrease in the level of inflammation after infection may be due to the reduced bacterial load. Thus, eliminating the load of leptospirosis in the treatment of early leptospirosis is the key.

Macrophages are the main immune cells involved in the clearance of leptospirosis in the host [26–28]. Interestingly, macrophages have significant phenotypic plasticity and can be reprogrammed in response to various environmental cytokines and pathogens to form an immune memory [29,30]. Intraperitoneal injection of LPS causes macrophages disappearance reaction [31,32]. At this time, Macrophages gather in the peritoneal lining and undergo a series of activation signals, leading to inflammation and the production of fully activated macrophages, resulting in up-regulation of macrophage function [33]. Recently, studies have shown that acute immune stimulation of LPS leads to transient changes in the abundance, composition, offspring and gene expression of hematopoietic stem cells, and enhances the response of related immune genes to secondary stimulation [34]. In addition, LPS can induce cell apoptosis in vivo. The immature macrophages in the engulf the apoptotic cells and thus become activated [35]. These activated macrophages showed stronger phagocytosis and bactericidal when the host was infected with *Leptospira*. The effect of LPS on central and peripheral immunity may be one of the mechanisms by which it plays a protective role.

In this experiment, we found that pre-treatment with LPS can fire a strong immune response to *Leptospira* in time. Moreover, LPS can enhance the bactericidal activity of macrophages. These findings will contribute to a better understanding of the pathogenic mechanism of leptospirosis and reveal new treatment strategies.
